# Effect of periodontal treatment in patients with periodontitis and diabetes: systematic review and meta-analysis

**DOI:** 10.1590/1678-7757-2019-0248

**Published:** 2019-12-17

**Authors:** Mauricio Baeza, Alicia Morales, Carlos Cisterna, Franco Cavalla, Gisela Jara, Yuri Isamitt, Paulina Pino, Jorge Gamonal

**Affiliations:** 1 Universidad de Chile, Facultad de Odontología, Departamento de Odontología Conservadora, Santiago, Chile.; 2 Universidad de Chile, Facultad de Medicina, Escuela de Salud Pública, Santiago, Chile.; 3 Universidad de Chile, Facultad de Odontología, Centro de Epidemiología y Vigilancia de las Enfermedades Orales (CEVEO), Santiago, Chile.; 4 Universidad de Chile, Facultad de Odontología, Departamento de Prótesis, Santiago, Chile.

**Keywords:** Periodontitis, Cardiovascular diseases, Diabetes mellitus

## Abstract

The evidence is inconclusive regarding the effect of periodontal treatment on glycemic control and systemic inflammation in patients with type 2 diabetes (T2D) and periodontitis

**Objective:**

To evaluate the effect of scaling and root planing (SRP) on the metabolic control and systemic inflammation of patients with type 2 diabetes (T2D).

**Methodology:**

A literature search was conducted using the MEDLINE database via PubMed and the Cochrane Central Register of Controlled Trials, from their oldest records up to July 2018. Only randomized clinical trials (RCT) were considered eligible for evaluating the effect of periodontal treatment on markers of metabolic control [glycated hemoglobin (HbA1C)] and systemic inflammation [C-reactive protein (CRP)] in patients with T2D. The quality of the studies was evaluated using the Cochrane Collaboration risk assessment tool. Meta-analyses were performed for HbA1c and CRP using random effects models. The size of the overall intervention effect was estimated by calculating the weighted average of the differences in means (DM) between the groups in each study. Heterogeneity was assessed using the Q-statistic method (x^2^ and I²). The level of significance was established at p<0.05.

**Results:**

Nine RCT were included. SRP was effective in reducing HbA1c [DM=0.56 (0.36-0.75); p<0.01] and CRP [DM=1.89 (1.70-2.08); p<0.01]. No heterogeneity was detected (I^2^=0%, p>0.05).

**Conclusions:**

SRP has an impact on metabolic control and reduction of systemic inflammation of patients with T2D.

## Introduction

Type 2 diabetes (T2D) and periodontitis are closely related noncommunicable diseases (NCDs) that present a high prevalence in the world, seriously compromising the quality of life of the affected population.^1^^,^^2^ The number of people with diabetes has increased from 108 million in 1980 to 422 million in 2014 and is considerably higher in middle and lower income countries.^3^ Diabetes has become a leading cause of death and disability in the region of the Americas, and if current trends continue, the burden of the disease will increase substantially over the next two decades.^4^ In Mexico and in the majority of the countries of Central and South America and the Spanish-speaking Caribbean, diabetes prevalence has been reported between 8% and 10%.^4^

In Chile, according to the last National Health Survey,^5^ the prevalence of diabetes is 12.3%. In turn, the prevalence of periodontal destruction in Chile is greater than 85% in people over 35 years of age.^6^ Despite the heterogeneity in the operational definition of the disease, most studies show that the prevalence of periodontitis in Latin America is high.^7^

Although there are no epidemiological studies of simultaneity of both pathologies, a meta-analysis showed that people with T2D have twice the risk of developing periodontitis compared with those without diabetes.^8^ These data show that almost all patients with T2D have periodontitis simultaneously.

T2D is characterized by an altered hyperglycemic metabolic state and is associated with a decrease in life expectancy of ten years and with long-term complications that may include cardiovascular disease, diabetic retinopathy, and kidney failure.^9^ Periodontitis, however, is currently considered a noncommunicable chronic inflammatory pathology of infectious etiology. The bidirectional pathogenic association between both diseases has been extensively documented.^10^ Diabetic patients are more susceptible to severe periodontitis and it may increase the risk of poor glycemic control.^11^ The effect of periodontitis on diabetes may be related to the penetration of the host tissues by bacteria or their degradation products into the systemic circulation. Activation of an exaggerated systemic inflammatory response to subgingival bacteria leads to an acute phase protein burst and systemically elevated levels of proinflammatory mediators which facilitate insulin resistance.^12^^,^^13^ Metabolic dysregulation in diabetes as a result of prolonged exposure to chronic hyperglycemia can lead to the glycosylation of proteins and lipids, called advanced glycation end- products (AGEs). They can explain many of the sequelae of diabetes, such as microvascular complications. Local periodontal tissue destruction may be a consequence of an exaggerated monocytic inflammatory response induced by AGEs accumulation and result in exaggerated secretion of local and systemic mediators leading to severe periodontitis.^14^ The binding of AGEs to monocyte receptors induces production of interleukin- 1 (IL-1), IL-6, insulin- like growth factor-1, tumor necrosis factor- alfa (TNF-α) and platelet- derived growth factor.^15^ Those are some factors that initiate and maintain an inflammatory response and regulate the transcription of human Acute Phase Reactants, like C-reactive protein (CRP). CRP is a sensitive inflammatory marker and an independent predictor of cardiovascular diseases.^16^

The poor glycemic control of diabetic patients, defined as glycated hemoglobin (HbA1c) values >7%, is associated with microvascular and macrovascular complications.^17^ Macrovascular complications also known as cardiovascular diseases (CVD) are an important cause of morbidity and mortality among individuals with T2D. Patients with T2D have an increased risk of premature atherosclerotic plaque development, which is the central pathogenic mechanism of CVD. To a great extent, the endothelial dysfunction present in patients with poor glycemic control is associated with the high production of CRP as a result of systemic inflammation caused by AGEs.^18^ The role of systemic inflammation and, consequently, of high levels of CRP has been described in all phases of atherosclerosis, from the onset and build-up of plaque to rupture. From the biological point of view, CRP participates in the atherogenic process, and its concentration predicts cardiovascular events.^19^ The cardiovascular effects of CRP have been described mainly through endothelial dysfunction by reducing nitric oxide levels. Endothelial dysfunction facilitates the activation, migration, and lodging of leukocytes inside the *tunica intima*. This phenomenon contributes to the formation of vascular lesions that are the basis of the development of atherosclerosis. When low-density lipoproteins reach a certain concentration threshold in the blood, they penetrate the interior of the arterial wall, where they are modified by oxidation processes. CRP binds to these lipoproteins (both native and oxidized) and facilitates phagocytosis and internalization mediated by the macrophages of the intima, promoting the formation of foam cells (macrophages with large amounts of oxidized lipids inside). The accumulation of foam cells is determinant for the pathogenic evolution of an arterial plaque, making it more prone to erosion or rupture and releasing its thrombogenic content. This prothrombotic property also adds to the ability of CRP to induce the production of tissue factor (initiator of the coagulation cascade) by activated macrophages.^20^ Also, CRP levels are elevated in patients with periodontitis^21^^.^^22^ and their CRP levels may go down after periodontal therapy.^21^

Studies conducted in the last decade indicate that the control of chronic inflammatory processes caused by periodontitis may constitute a new approach in reducing the risk of cardiovascular complications in patients with T2D. Based on this concept, clinical trials have been designed to determine the possible beneficial effects of conventional periodontal treatment, consisting of the mechanical removal of the subgingival biofilm by means of scaling and root planing (SRP) in diabetic patients. Despite the variability, the results of these studies have been mostly favorable. However, systematic reviews have been limited to the analysis of HbA1c as an end point measure,^23^ without considering other variables such as those associated with systemic inflammation that can provide us with a better approach to reducing the risk of diabetic complications, especially those related to CVD. The objective of this systematic review was to answer the following question: Can conventional periodontal treatment compared with the absence of periodontal treatment improve metabolic control and reduce systemic inflammation in patients with periodontitis and T2D?

## Methodology

### Protocol

The protocol was designed in accordance with the Cochrane standards for systematic reviews. The search criteria complied with the Preferred Reporting Items for Systematic reviews and Meta-Analysis Protocols (PRISMA) guidelines.

### Criteria for the inclusion of studies

Type of studies: The study design was a randomized controlled clinical trial, as it is the most appropriate type of study to answer the research question since it presents a lower risk of systematic errors and greater control over potential confounding variables, constituting the best scientific evidence to support the efficacy of therapeutic interventions.

Type of participants and inclusion/exclusion criteria: Participants with a diagnosis of T2D and periodontitis were included. Other inclusion criteria were the following: (1) Participants with 3 or more months of post intervention follow-up and (2) HbA1c and CRP evaluation at the beginning and end of the follow-up.

Types of periodontal interventions: Periodontal interventions were based on different conventional treatments, including oral hygiene instruction and SRP (with or without flap surgery). Periodontal interventions were compared with the passive option (without periodontal treatment).

Outcomes:

Primary: HbA1C and CRP

Secondary: Occurrence of adverse events related to periodontal treatment.

### Search strategy

We searched the following databases from their oldest records until July 10, 2018: 1) MEDLINE through PubMed and 2) Cochrane Central Register of Controlled Trials (CENTRAL). The articles in both databases are only available in English. The search terms (MeSH terms) were “periodontitis” OR “periodontal” AND (therapy OR treatment) AND (diabetes OR metabolic control OR glycemic control OR glycated hemoglobin OR HbA1c OR systemic inflammation OR C-reactive protein OR hsCRP).

### Study eligibility and data extraction

All retrieved articles were evaluated by two independent reviewers (MB and AM). Inter reviewer reliability in the study selection process was determined by the Cohen *k* test, assuming an acceptable threshold value of 0.61.^24^^,^^25^ Disagreements were resolved by consensus. If a disagreement persisted, the judgment of a third reviewer (JG) was considered final. Irrelevant records (abstracts not available, bibliographic reviews, descriptive studies, animal studies) were excluded, and the full texts of potentially relevant studies were examined to answer the question of interest. The relevant studies that met the inclusion criteria were analyzed, with the following data extracted: 1) demographic characteristics (age, sample size, country where the study was conducted), 2) definition of disease, 3) control measures of T2D, 4) periodontal intervention, 5) outcome variable (HbA1c and/or CRP) and their initial values, 6) follow-up period and final values, and 7) results (average change in HBA1c and/or CRP in each group).

### Quality of the evidence

The evaluation of the quality of the selected studies was carried out using the Cochrane Collaboration tool to assess the risk of bias.^26^

### Diagnostic evaluation

T2D was diagnosed according to the criteria of the World Health Organization,^27^ defined as fasting plasma glucose >126 mg/dl and/or impaired oral glucose tolerance test at 2 h. Due to the heterogeneity of the criteria and the definitions of periodontitis, any diagnosis of periodontitis was accepted as defined by the authors.

### Statistical analysis

The data extracted from the studies were analyzed using RevMan 5.3 software (The Nordic Cochrane Centre, The Cochrane Collaboration, Copenhagen, Denmark). The corresponding meta-analyses were carried out to determine the effect of the periodontal intervention on the variables HbA1c and CRP, comparing the group that received the periodontal treatment (intervention group) with the group that did not receive periodontal treatment (control group). The effect size was estimated and reported as the difference between the intervention group and the control group in the means of the changes occurring in the levels of HbA1c and CRP between the start and the end of the follow-up, and the 95% confidence interval (CI) was calculated. Due to the expected heterogeneity between the studies, a random effects model was used.^28^ The combined overall effect (weighted average) was considered significant if *p*<0.05. The forest plots for each meta-analysis present raw data (means, standard deviations, and sample sizes), point estimates (shown as blocks) and CIs (shown as lines) of the effect of each study, the total number of participants *per* group, the general average effect of the model (Z statistic), and the weight percentage for each study. Heterogeneity was assessed using the Q-statistic method based on x^2^ and the I^2^ measurement. The publication bias of the articles was investigated for each outcome variable (HbA1c and CRP) by visual inspection of asymmetries in the funnel plot.^29^

## Results

### Search results

A total of 402 articles were retrieved using databases from two online sources (MEDLINE and Cochrane Library). After the elimination of duplicates (n=53), 349 titles and abstracts were reviewed, 292 of which were excluded because they did not meet the basic inclusion criteria (designs without intervention, reviews, or patients with DT1) (inter- reviewer agreement, k=0.76). Fifty-seven complete articles were evaluated in more detail to determine their eligibility, nine of which met the criteria for inclusion in the systematic review and meta-analysis (inter reviewer agreement, k=0.88). The flow chart following the PRISMA criteria and model of the complete search and inclusion process of the studies is illustrated in Figure 1.

**Figure 2:**
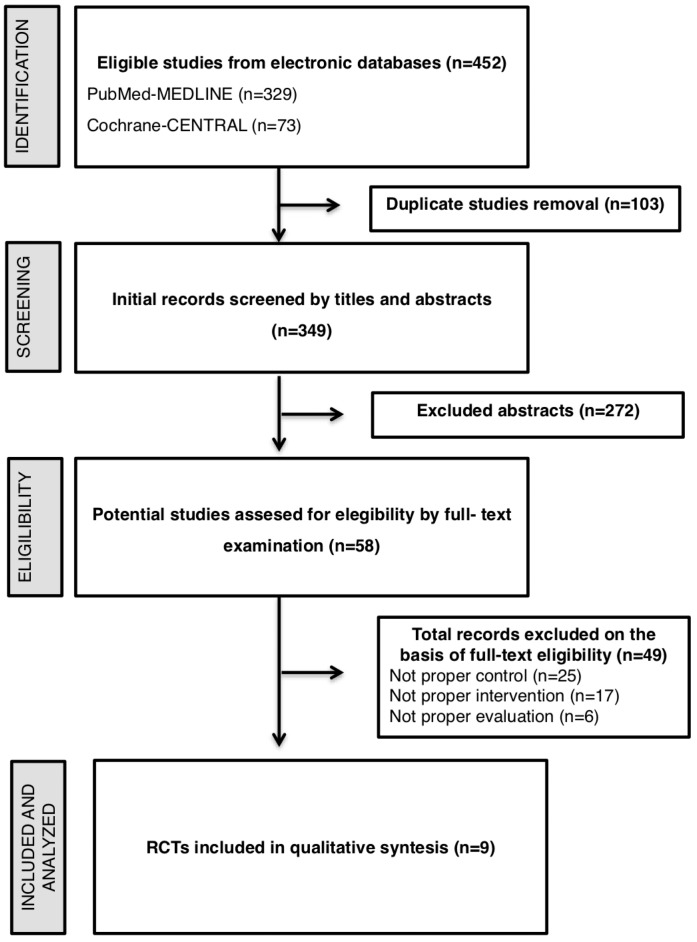
Selection of studies for systematic review

### Description of the studies

The main characteristics of the included studies (n=9) are presented in Figure 2. All the included studies^30^^–^^37^ were randomized clinical trials with a control group without periodontal intervention. To control for the possible placebo effect, oral hygiene instruction (IHO) was administered in two studies;^31^^–^^36^ supragingival scaling was performed in another study in the control group;^34^ and no type of treatment was reported for the control group in the remaining studies. The intervention in all the studies was a conventional periodontal treatment with nonsurgical (SRP), except for the study by Chen, et al.^30^ (2012), which included surgical debridement at 3 months. The definition used for the diagnosis of periodontitis varied widely among the studies. Periodontitis was diagnosed in four studies according to the criteria of the American Academy of Periodontology (AAP),^30^^,^^34^^,^^35^^,^^37^ in one study^36^ according to the Eke criteria,^38^ in another study according to unspecified criteria,^33^ and in the remaining three studies according to different severity criteria.^32^^,^^37^^,^^39^ Regarding smoking, one study excluded smokers,^37^ another did not report whether smokers were included as a selection criterion,^39^ and the rest of the studies included smokers, with a balance between the groups after randomization. In relation to T2D, all the studies reported the diagnostic criteria following the WHO guidelines, with a variable onset date. In turn, all reported metabolic control measures for the treatment of T2D, except one,^39^ and all reported that there were no changes in the T2D treatment scheme during follow-up, except one that did not report these data.^35^ The initial values, final values, and changes (delta) in HbA1c and/or CRP in each group of each study are presented in Figure 1.

**Figure 2:**
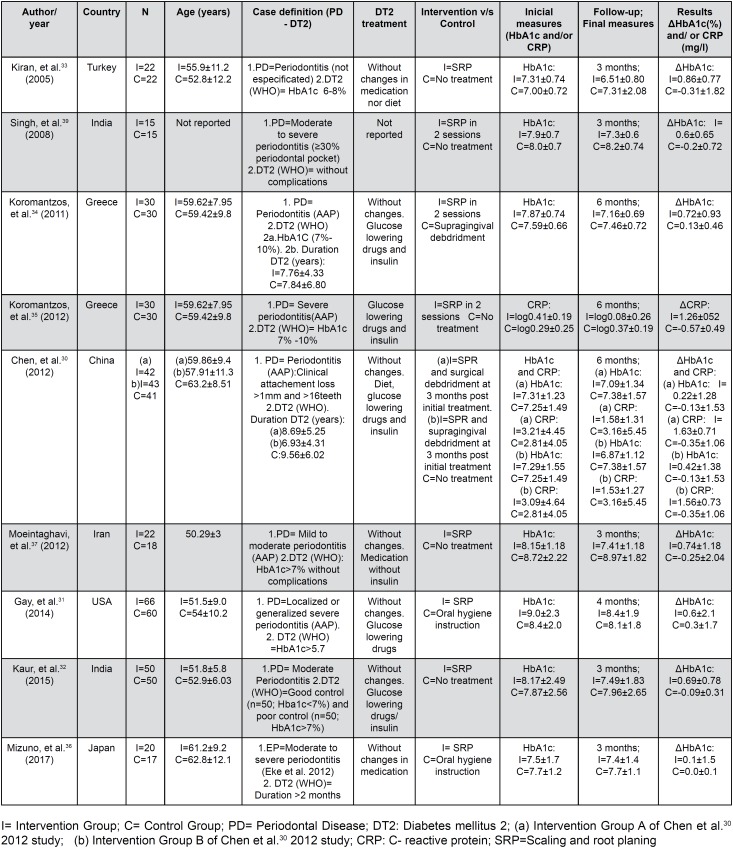
Characteristics of studies included in the systematic review

The main reasons to exclude 49 studies were “inadequate control group” (n=25),^40^^–^^64^ “inadequate intervention and/ or inadequate case definition” (n=17),^65^^–^^81^ “inadequate evaluation and or high risk of bias” (n=6)^82^^–^^87^ (Figure 7).

### Risk of bias in the included studies

**Figure 3:**
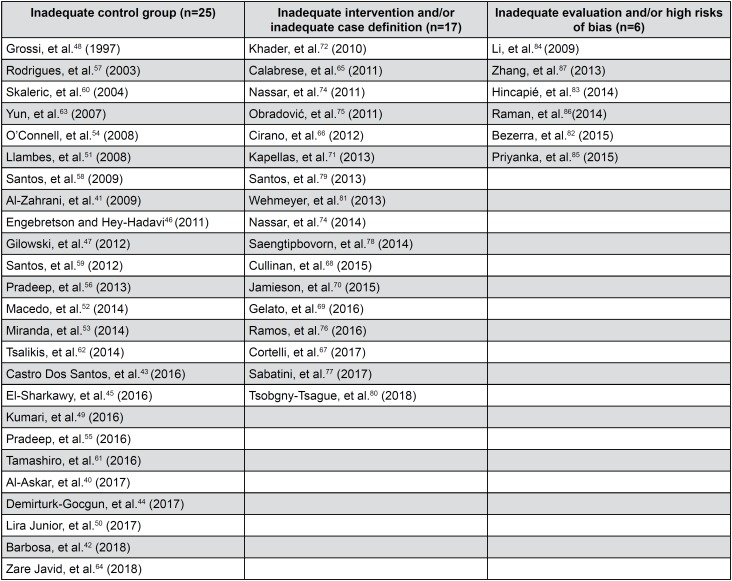
Characteristics and names of the excluded studies (n=49)

**Figure 4:**
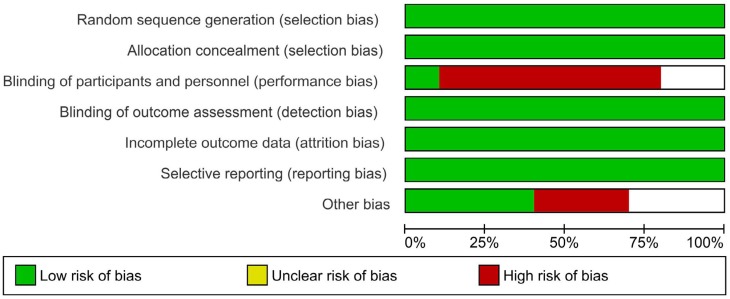
Risk of bias graph: review author's judgment about each risk of bias item presented as percentages across all included studies

To determine the validity of the included studies, a tool developed by the Cochrane Collaboration was used to assess the risk of bias in clinical trials.^26^ Using this tool, the reviewer evaluated the risk of bias of the selected studies using the following parameters: random sequence generation, allocation concealment, blinding of participants and personnel, analysis intention (blinding of outcome assessment), incomplete outcome data, selective reporting (selection of the reported results), and other types of bias not considered previously (e.g., design bias, contamination bias). All articles adequately reported random sequence generation, allocation concealment, blinding of outcome assessment, incomplete outcome data and selective reporting. In six articles in which the control group did not undergo any procedure, it was not possible to blind the personnel and the participants; only in one study^34^ was it possible to ensure the blinding of the participants, and blinding in the other two studies was unclear.^31^^,^^69^ Other unspecified types of bias were also considered as associated with the lack of information regarding control measures and possible changes in the management of T2D^39^ or interventions performed by a general dentist and nonspecialist,^36^ and there were less clear situations such as those related to the contamination of the results (contamination bias) due to the effect of procedures in the control group (debridement and/or IHO). The general scheme of the risk of bias is presented in Figure 3, and a detailed summary by study is provided in Figure 4.

### Effect of the intervention

Primary HbA1c Outcomes: The results of the meta-analysis indicate a significant reduction in %HbA1c from the beginning to the end of the follow-up in the intervention group [differences in means (DM)=0.56, 95% CI (0.36-0.75), *p*<0.00001]. In this meta-analysis, no heterogeneity was detected among the included studies (x^2^=5.89, I^2^=0%, *p*=0.66) (Figure 6A).

**Figure 5:**
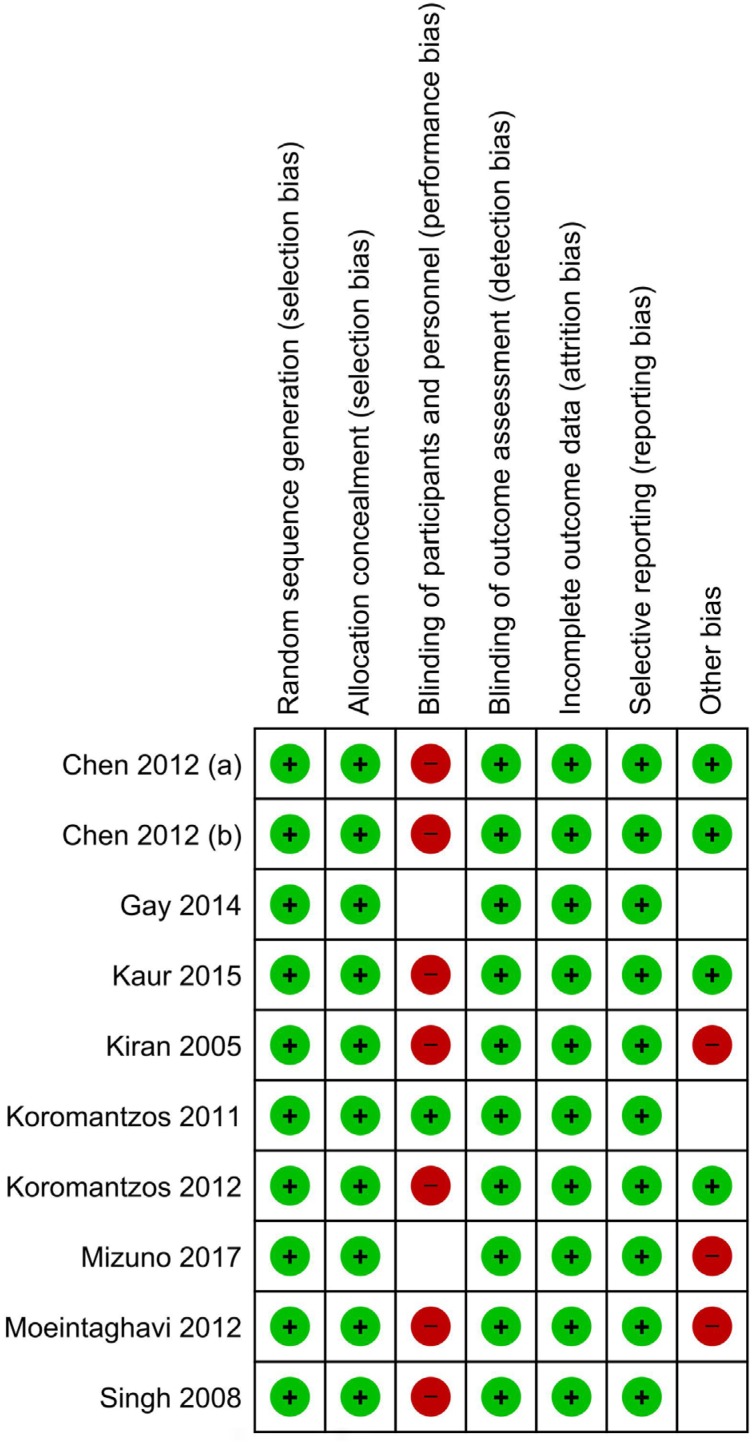
Risk of bias summary: review author's judgments about each risk of bias item for each included study

**Figure 6:**
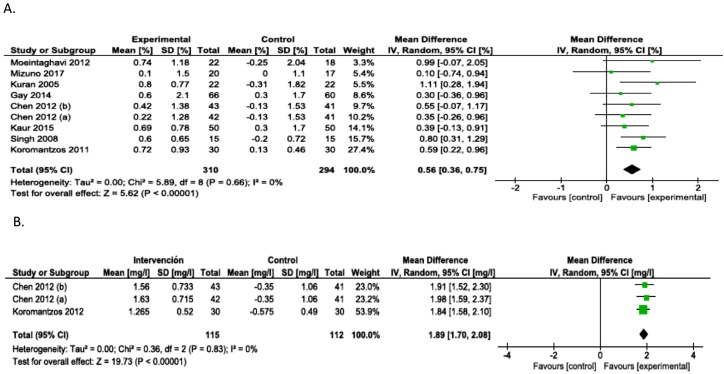
Forest Plot of comparision: A. Periodontal therapy outcome: Change in HbA1c (%HbA1c). B. Periodontal therapy outcome: Change in CRP

Primary CRP Outcomes: Regarding the results of CRP levels, the meta-analysis indicated a significant reduction from the start to the end of follow-up in favor of the intervention group [DM=1.89, 95% CI (1.70-2.08), *p*<0.00001]. No heterogeneity was detected among the included studies (x^2^=0.36, I^2^=0%, *p*=0.83) in this meta-analysis (Figure 6B).

Secondary Outcome: None of the included studies reported the occurrence of adverse effects or complications related to periodontal treatment.

### Publication biases

**Figure 7:**
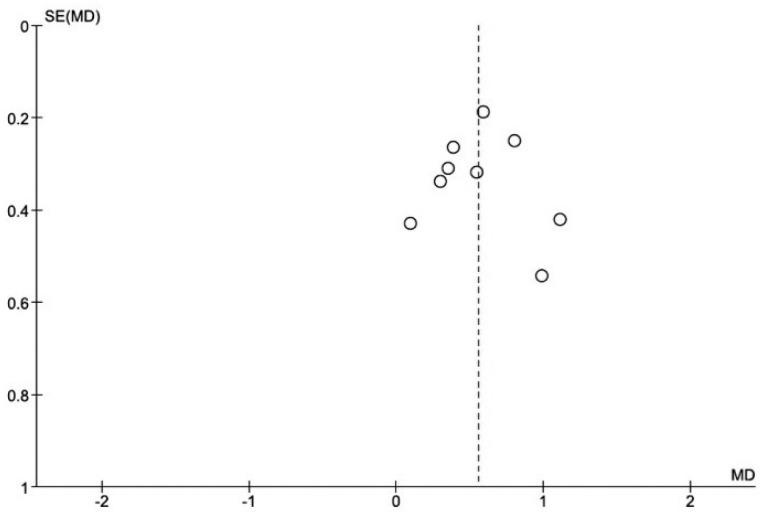
Funnel plot of comparision: Periodontal therapy outcome: Change in HbA1c

Visual inspection of the funnel plot for studies included in the meta-analysis that evaluated the effect of periodontal treatment on HbA1c levels does not suggest the presence of publication biases (Figure 7). Regarding the studies included in the CRP meta-analysis, the limited study number does not allow a clear assessment of the absence of possible publication bias.

## Discussion

The present systematic review with its two meta-analyses indicates that conventional periodontal treatment can improve metabolic control and reduce systemic inflammation in patients with T2D by reducing serum levels of HbA1c and CRP, respectively. The results of the metabolic control achieved by treatment and the reduction of systemic inflammation are consistent with the results of other systematic reviews and meta-analyses.^88^^–^^90^ However, in the present review, for the first time, the effect of the periodontal intervention on both outcome variables was evaluated simultaneously, with the same study methodology and inclusion criteria. Thus, this review used strict eligibility criteria, such as the presence of ≥3 months of follow-up, which reflects the period in which the changes in HbA1c and CRP are more stable and relevant. In addition, randomized studies comparable to each other were included, which strongly contributed to the reliability of the results.

The meta-analysis determining the overall effect of periodontal treatment on HbA1c levels showed relative consistency between the studies included, an absence of heterogeneity (I^2^=0%), and a significant effect in the intervention group (*p*<0.01). These results, similar to those previously found, reinforce the hypothesis that suggests that periodontal treatment generates improved HbA1c levels in patients with T2D. The effect size, measured in terms of mean differences between the delta (initial-final) of the intervention group vs. control, showed an overall effect of 0.56% (0.36-0.75) at the end of the follow-up period. The clinical implications of achieving better metabolic control of diabetic patients are clearly established; specifically, the microvascular complications related to diabetes are reduced by 35% for each 1% decrease in HbA1c levels. Additionally, an absolute decrease of 1% in the HbA1c level can decrease the risk of any death related to diabetes by 21%.^17^ Future studies are necessary to determine the direct impact of periodontal treatment on complications and the risk of dying associated with T2D.

Although these results (0.56%) are consistent with previous studies, it is interesting to see that the overall effect found in this study considerably exceeds the 0.29% HbA1c reported by Simpson, et al.^89^ (2015). It is likely that the incorporation of studies subsequent to Simpson's review, such as the study by Kaur, et al.^32^ (2015), will favor the results of the present study. Another major explanation is that in the studies included in the present review the interventions were based on conventional periodontal treatment that included: oral hygiene instruction and SRP (with or without flap surgery) compared with the passive option (without periodontal treatment), these results were similar to those reported by Simpson, et al.^89^ (2015) with a reduction of 0.41% HbA1c when similar conditions were analyzed. However, when Simpson analyzed different interventions, for example, when incorporating antimicrobials or alternative periodontal treatments, the results were considerably lower^89^.

Regarding CRP levels, although the meta-analysis showed a significant overall effect with an absence of heterogeneity, this result must be evaluated with caution due to the small number of studies analyzed. However, the results are consistent with each other and with other studies^88^ that reflect the impact of periodontal treatment at the marker-level of systemic inflammation. This result complements those found in previous systematic reviews in relation to the potential effects of periodontal treatment on the general health of patients with T2D, enhancing the hypothesis regarding a dual explanatory path for reducing systemic inflammation, namely, one path directly through the reduction of proinflammatory mediators such as TNF-alpha, IL-1, and IL-6, which induce acute-phase responses with hepatic biosynthesis and elevated serum CRP levels, and another path indirectly through the reduction of HbA1c itself as a result of the reduction of insulin resistance. In this case, the effect size was a mean difference of 1.89 mg/l [(1.70-2.08), *p*<0.01]. CRP is the most commonly used inflammatory marker in clinical practice, and its importance not only lies in its reliability but also, along with some other acute-phase reactants, in its ability to be an active part of the pathogenic mechanism of atherosclerosis.^91^ Prospective studies show that increased CRP plasma concentrations of small magnitude, even within limits that were previously considered “normal” (below 10 mg/L), predict acute cardiovascular events apart from other risk factors.^92^ Patients with T2D present an increase in systemic inflammation, which partly explains the considerable increase in cardiovascular risk. This increase in inflammation adds to the presence of chronic inflammatory processes such as periodontitis, which constitutes an additional source of systemic proinflammatory mediators, and could explain the even greater increase in cardiovascular risk.

The included studies considered different levels of severity and extent of periodontitis (moderate, moderate to advanced, severe) that could have an impact on the final outcome (systemic and local). More severe pathologies could generate a more significant change in the local and systemic inflammation of patients with T2D, which could partly explain some differences in the results. Similarly, the included studies presented differences in their criteria for selecting diabetic patients with different degrees of metabolic control. Increased changes in HbA1c levels after periodontal treatment have been observed in patients with poor metabolic control and/or patients with acceptable or optimal control of HbA1c levels (<7%).^32^ When stratifying by level of control, it was observed that the deltas (initial-final) in the periodontal intervention group were 1.49±0.98% in patients with poor control (HbA1c<7%) and 0.16±0.32% in patients with good metabolic control. Quintero, et al.^93^ (2018) reported that periodontal treatment generated a greater impact in the reduction of HbA1c in patients with levels >9% (0.31% vs 0.88%).^93^ These aspects must be considered for future research and for an adequate interpretation of the results.

Regarding the excluded studies, 272 studies were excluded in a first phase of screening. Since this phase, differences have already been observed with Simpson's Cochrane review,^89^ which unlike ours includes patients with type 1 diabetes. Later in the eligibility stage, 48 complete articles were excluded due to inadequate control group (n=25), inadequate intervention and/or inadequate case definition (n=17) and inadequate evaluation and/or high risks of bias (n=6). There are clearly differences with the Simpson's review who uses different modalities of periodontal treatment (with the use of antimicrobials or new therapies) not included in the present review. Other articles such as Zhang, et al.^87^ (2013) or Li, et al.^84^ (2011) for presenting an inadequate evaluation and high risks of bias were also excluded from the present review. This may explain why the Simpson's Cochrane review includes more articles (n=35) and excludes less (n=12) unlike our review that includes less (n=9), and excludes more (n=49). Our purpose was to be highly rigorous when including the studies for the present systematic review. More details of the characteristics and names of the excluded studies are presented in Figure 3.

The quality of evidence from a systematic review depends to a large extent on the quality of the included studies. In this case, the eligible study design was the randomized controlled clinical trial that, due to its lower risk of bias, represents the most appropriate design for assessing the efficacy of periodontal treatment. However, it is necessary to clarify that the type of study does not necessarily guarantee a good quality of evidence and that, therefore, clinical trials may also present biases that prevent the attainment of accurate results, invalidating our results. In this case, the included studies presented some risks of important biases that included performance bias due to the inherent nature of the design and the lack of an adequate placebo. Regarding other sources of bias, generally, the included studies are of good quality because they have low risks of systematic errors.

Another limitation of this study was the lack of a statistical analysis to evaluate the possibility of publication bias (such as the Egger test) that could support the funnel plot results. In addition, it should be noted that only two database sources were used for the bibliographic search; since other databases exist, it is important for future reviews to include additional sources of information. Finally, only English language literature was reviewed. Searching vernacular indexed literature might give more data for systematic review.

The high prevalence, morbidity and mortality of T2D explain why the management of the disease and its complications consume such a high percentage of the health budget of different countries. While in Chile it reaches 10.2%,^94^ in Mexico it exceeds 15% of the total health budget. In 2014, DM2 caused 4.9 million deaths and an expenditure of 612 billion dollars worldwide.^95^

The results of the present systematic review with its two meta-analyses reinforce and complement the evidence regarding the efficacy of periodontal treatment in the improvement of metabolic control and in the reduction of systemic inflammation of patients with T2D. In this context, the implication of incorporating periodontal treatment into the care of patients with T2D could constitute a policy of high impact for the public health of the country.

## Conclusion

SRP has an impact on metabolic control and reduction of systemic inflammation of patients with T2D. Periodontal treatment could constitute an innovative therapeutic approach as a public health measure to reduce complications and improve the cardiovascular health of patients with T2D. Intervention and cost-effectiveness studies in the context of public health systems in different countries are necessary to evaluate the possible design of public policies in this area.
